# 1853. Relevance of *Actinomyces* Bacteremia

**DOI:** 10.1093/ofid/ofac492.1482

**Published:** 2022-12-15

**Authors:** Mamta Sharma, Jonathan T Arcobello, Farah Tanveer, Sanjay Revankar, Asish Bhargava

**Affiliations:** Ascension St John Hospital & Medical Center, Grosse Pointe Woods, MI. 48236, Michigan; Wayne State University, Detroit, Michigan; Ascension St John, Grosse Pointe Woods, Michigan; Wayne State University, Detroit, Michigan; Ascension St John, Grosse Pointe Woods, Michigan

## Abstract

**Background:**

*Actinomyces* species are Gram-positive anaerobic bacilli colonizing the human oropharynx, gastrointestinal tract, and urogenital tract asso­ciated with a wide range of infections. The isolation of *Actinomyces* spp. from sterile clinical samples is regarded as significant. We reviewed the risk factors, clinical features, and outcomes in patients with *Actinomyces* bacteremia.

**Methods:**

We conducted a retrospective study of all inpatients with *Actinomyces* bacteremia from two tertiary care centers from 1/1/06 -9 /26/19 . Data were collected on demographic and clinical characteristics, co-morbidities, primary source of infection, treatment received and duration of therapy and outcomes. True bacteremia was defined as *Actinomyces* bacteremia with systemic manifestations of infection.

**Results:**

A total of 82 cases of positive blood cultures were identified of which 33 were true bacteremia, based on clinical criteria among 19 females and 14 males ranging from 19-93 years (63.8 ±19.5). Majority of patients were blacks (70%). Clinical risk factors predominantly were diabetes mellitus (21%), chronic renal failure (18%) and active malignancy (12%). Majority of blood cultures were positive within 48 hrs. of admission 84.8%. Skin and soft tissue (27%) was the most common source followed by respiratory (15%), intrabdominal (15%), osteomyelitis (12%), odontogenic (6%), endovascular (6%) genitourinary (3%) and unknown source (16%). In 27 (81%) cases, bacteremia was isolated to species level: *A.meyeri* 8 (30%), *A.odontolyticus* 7 (26%)*, A.israelli* 7 (26%), *A.turicensis* 4 (15%) and *A.neuii* 1 (4%). The infectious diseases service was consulted in 20 cases of true bacteremia. Duration of antibiotics ranged between 0-84 days (17.6 ±20.7). All-cause mortality during hospitalization was 24% (8).

**Conclusion:**

Not all *Actinomyces* bacteremia may be relevant and can represent transient bacteremia. Better awareness and involvement of infectious disease service is recommended in understanding of the clinical significance, to ensure appropriate therapy for patients thereby implementing antibiotic stewardship to improve antibiotic use and, patient safety and improve outcomes. Further research will help to identify the true significance of these isolates.

**Disclosures:**

**All Authors**: No reported disclosures.

